# Modulation of the photobehavior of gefitinib and its phenolic metabolites by human transport proteins

**DOI:** 10.3389/fphar.2024.1387057

**Published:** 2024-05-16

**Authors:** Lorena Tamarit, Meryem El Ouardi, Emilio Lence, Inmaculada Andreu, Concepcion González-Bello, Miguel A. Miranda, Ignacio Vayá

**Affiliations:** ^1^ Departamento de Química/Instituto de Tecnología Química UPV-CSIC, Universitat Politècnica de València, Valencia, Spain; ^2^ Unidad Mixta de Investigación UPV-IISLaFe, Hospital Universitari i Politècnic La Fe, Valencia, Spain; ^3^ Centro Singular de Investigación en Química Biolóxica e Materiais Moleculares (CiQUS), Departamento de Química Orgánica, Universidade de Santiago de Compostela, Santiago de Compostela, Spain

**Keywords:** anticancer drugs, fluorescence, metabolites, molecular dynamics, protein binding constants

## Abstract

The photobiological damage that certain drugs or their metabolites can photosensitize in proteins is generally associated with the nature of the excited species that are generated upon interaction with UVA light. In this regard, the photoinduced damage of the anticancer drug gefitinib (GFT) and its two main photoactive metabolites GFT-M1 and GFT-M2 in cellular milieu was recently investigated. With this background, the photophysical properties of both the drug and its metabolites have now been studied in the presence of the two main transport proteins of human plasma, i.e., serum albumin (HSA) and α1-acid glycoprotein (HAG) upon UVA light excitation. In general, the observed photobehavior was strongly affected by the confined environment provided by the protein. Thus, GFT-M1 (which exhibits the highest phototoxicity) showed the highest fluorescence yield arising from long-lived HSA-bound phenolate-like excited species. Conversely, locally excited (LE) states were formed within HAG, resulting in lower fluorescence yields. The reserve was true for GFT-M2, which despite being also a phenol, led mainly to formation of LE states within HSA, and phenolate-like species (with a minor contribution of LE) inside HAG. Finally, the parent drug GFT, which is known to form LE states within HSA, exhibited a parallel behavior in the two proteins. In addition, determination of the association constants by both absorption and emission spectroscopy revealed that the two metabolites bind stronger to HSA than the parent drug, whereas smaller differences were observed for HAG. This was further confirmed by studying the competing interactions between GFT or its metabolites with the two proteins using fluorescence measurements. These above experimental findings were satisfactorily correlated with the results obtained by means of molecular dynamics (MD) simulations, which revealed the high affinity binding sites, the strength of interactions and the involved amino acid residues. In general, the differences observed in the photobehavior of the drug and its two photoactive metabolites in protein media are consistent with their relative photosensitizing potentials.

## 1 Introduction

**FIGURE 1:**
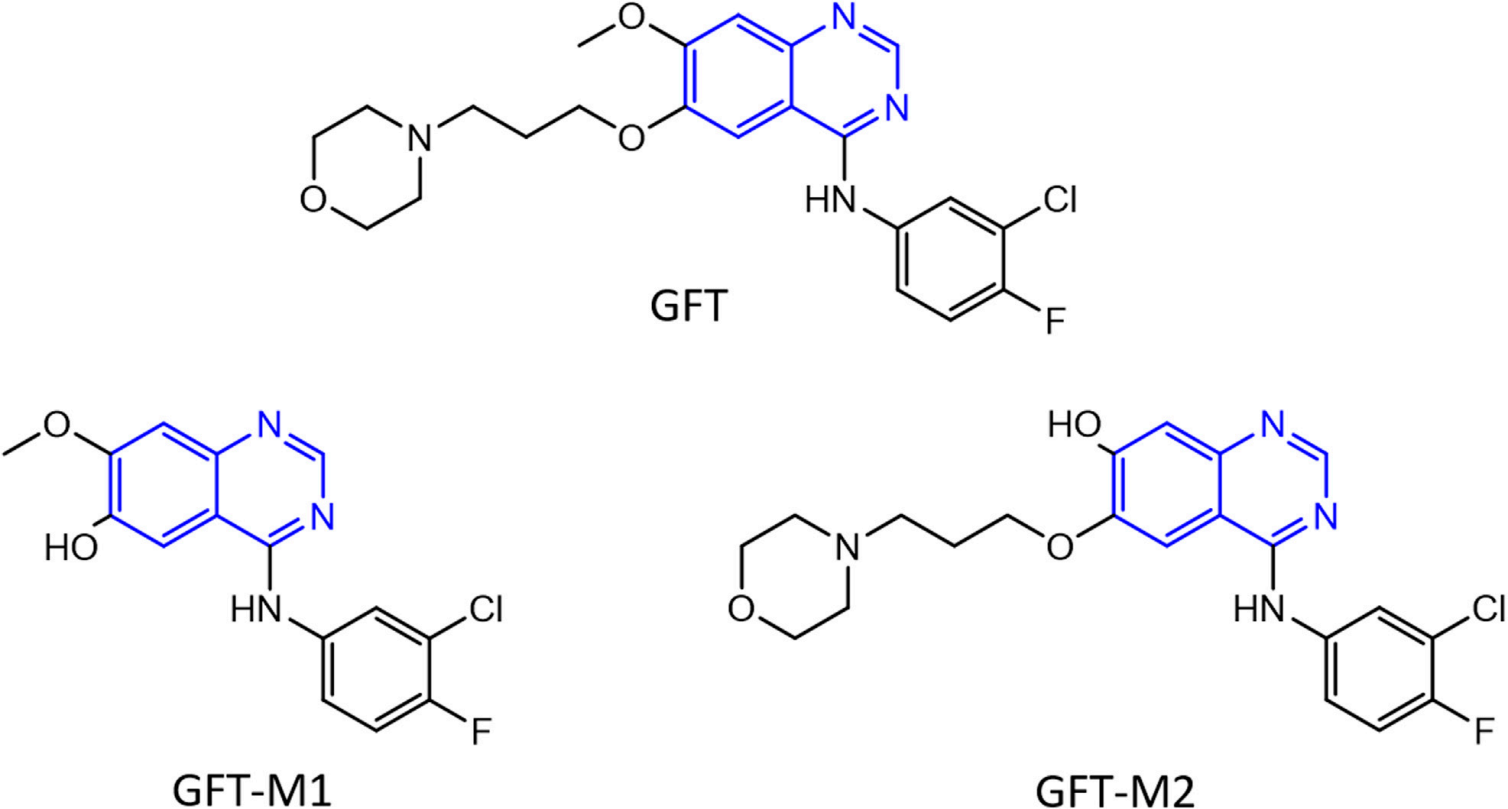
Chemical structure of gefitinib (GFT) and its *O*-desmorpholinopropyl and *O*-desmethyl metabolites GFT-M1 and GFT-M2, respectively. The quinazoline chromophore is highlighted in blue.

The binding of drugs to plasma proteins is involved in the modulation of relevant processes including drug pharmacokinetics (*i.e.,* absorption, distribution, metabolism and elimination) and pharmacodynamics (pharmacological effects) (Krasner, 1972; Kragh-Hansen et al., 2002; Vuignier et al., 2010). This binding is usually reversible, with an equilibrium between bound and free drug. In this regard, it is commonly stated that only unbound drugs are pharmacologically and toxicologically active since they can cross membrane barriers to be distributed to tissues (Lindup and Orme, 1981). However, photochemically active drugs can induce damage to biomolecules after absorption of solar light, which is generally associated with phototoxicity and photoallergy (Quintero and Miranda, 2000; Stein and Scheinfeld, 2007; Vayá et al., 2014; Monteiro et al., 2016; Blakely et al., 2019; Kowalska et al., 2021). Hence, the interaction of photoactive compounds with plasma proteins must have a strong influence on drug biological effects, beyond the transport and the intrinsic photochemical properties associated with the chemical structure of the drug. In this context, drugs containing the quinazoline moiety (highlighted in blue in Figure 1) are known to produce photodermatosis (Selvam and Kumar, 2011).

The photosensitizing potential associated with this moiety is well exemplified by gefitinib (GFT), which is an orally active first-generation tyrosine kinase inhibitor (TKI) (Solassol et al., 2019; Huang et al., 2020; Pottier et al., 2020; Cohen et al., 2021) clinically used for the treatment of lung cancer and locally advanced or metastatic non-small cell lung cancer (Cohen et al., 2003; Paez et al., 2004). GFT is metabolized via CYP3A4 to form a variety of derivatives (Hartmann et al., 2009) including those shown in Figure 1, *O*-desmorpholinopropyl gefitinib (GFT-M1) and *O*-desmethyl gefitinib (GFT-M2), which have recently revealed to be biologically photoactive (El Ouardi et al., 2023). Lapatinib (LAP), which is other TKI currently used for the treatment of lung and breast cancers (Lin et al., 2008; Medina and Goodin, 2008; Lin et al., 2009), is also a relevant example. In both cases, both the parent drug and their photoactive metabolites can induce damage in cellular milieu (García-Laínez et al., 2021; Tamarit et al., 2021; El Ouardi et al., 2023); the associated mechanism is related with the excited species that are formed upon irradiation of the supramolecular drug or metabolite@protein complexes with UVA light (Andreu et al., 2020; Vayá et al., 2020; Tamarit et al., 2021; Tamarit et al., 2022).

Human serum albumin (HSA) and human α_1_-acid glycoprotein (HAG) are the most abundant proteins in plasma. One of their main physiological functions is to transport a broad variety of drugs with sufficient affinity through the bloodstream for their selective delivery to specific targets (Trainor, 2007); generally, the binding affinity (*K*_*B*_) is in the range of 10^4^–10^6^ M^-1^ (Kremer et al., 1988; Carter and Ho, 1994; Peters, 1995; Huang and Ung, 2013). In particular, HSA is the most abundant protein in blood plasma, and contains multiple binding sites, *i.e.,* stie I, II and II, where acidic, neutral, and basic drugs can interact (Sudlow et al., 1976; Zsila, 2013). As regards HAG, it is a highly glycosylated protein that contains multiple binding sites, but generally drugs bind almost exclusively to a large and flexible cavity (Kremer et al., 1988; Maruyama et al., 1990). Its concentration is much lower than that of HSA, but since it is an acute-phase protein, its serum levels can be increased in certain disease states including inflammation, depression and cancer (Kremer et al., 1988). In this context, it has been previously proposed that high concentration of HAG can affect the pharmacodynamics of some drugs *in vivo* (Yoo et al., 1996; Holladay et al., 1998; Gambacorti-Passerini et al., 2003; Trainor, 2007).

Recent publications about the photobehavior of GFT and GFT-M1 within HSA correlate their photophysical properties with their photosensitizing potential (Tamarit et al., 2021; Tamarit et al., 2022). In this regard, selective excitation of the protein-bound drug results in emission from locally excited (LE) singlet states; their main features are to display maximum fluorescence at wavelengths *ca.* 390 nm with low quantum yields (ϕ_F_ ∼ 0.02) and short lifetimes (τ_F_ ∼ 1.3 ns). Noteworthy, the fluorescence profile of GFT@HSA is very similar to that observed in non-polar solvents such as cyclohexane, where formation of LE states has been demonstrated by means of ultrafast transient absorption spectroscopy. Concerning GFT-M1, although its fluorescence properties in organic solvents are comparable with those of GFT, remarkable differences are noticed after binding with HSA; here, excited state proton transfer (ESPT) to form phenolate-like species, which emit at longer wavelengths (λ_max_ ∼ 430 nm) and higher τ_F_ values (∼2.5 ns) than LE sates, is the predominant process (Tamarit et al., 2022). This result is supported by means of ultrafast spectroscopy and by molecular docking simulations. The photosensitizing damage from both GFT and GFT-M1 in the biological media is consistent with the involvement of a type I mechanism (Tamarit et al., 2021; Tamarit et al., 2022; El Ouardi et al., 2023).

In view of the importance of drug or metabolite@protein interactions, the binding of GFT and its two photoactive metabolites GFT-M1 and GFT-M2 with the main transport proteins in plasma has been investigated in the present work. To this end, spectroscopic techniques in the steady-state and time-resolved modes have been used. In this regard, fluorescence spectroscopy is a widely used analytical technique to study ligand@protein interactions due to its high sensitivity and capability to probe different microenvironments. Thus, the yield of transients formation in addition to their spectral profile and kinetics evolution may be strongly affected by the surroundings of the investigated ligand (*i.e.,* drug or metabolite) (Vayá et al., 2014). From the emission spectra and lifetimes of the excited species formed in the ligand@protein complexes, it has been possible to determine binding constants and the stoichiometry of the complex. In parallel, molecular dynamics (MD) simulations have also been done with the aim of investigating in atomic detail the binding of GFT, GFT-M1 and GFT-M2 within HSA and HAG, to achieve a better understanding of the experimental results. In this context, MD simulations has proven to be a powerful tool for studying the strength and conformational characteristics of the interactions of a drug with the amino acids located in the protein binding sites (Pérez-Ruiz et al., 2017; Spitaleri and Rocchia, 2019; Vayá et al., 2020). All these features are relevant since they can be directly connected with the photosensitizing potential recently reported for GFT and its metabolites GFT-M1 and GFT-M2 (El Ouardi et al., 2023).

## 2 Materials and methods

### 2.1 Chemicals and reagents

Gefitinib (GFT) and *O*-desmethyl gefitinib (GFT-M2) were purchased from Quimigen. *O*-Desmorpholinopropyl gefitinib (GFT-M1) was purchased from Fluorochem. *N*-Acetyl-L-tyrosine methyl ester (NAc-TyrMe), *N*-acetyl-L-tryptophan methyl ester (NAc-TrpMe), anthracene, human serum albumin (HSA) and α_1_-acid glycoprotein from human plasma (HAG) were purchased from Sigma-Aldrich. PBS Buffer was prepared by dissolving phosphate-buffered saline tablets (Sigma) using ultrapure water from a Millipore (Milli-Q Synthesis) system. Spectrophotometric solvents (acetonitrile, 1,4-dioxane, toluene and cyclohexane) were obtained from Scharlab and used without further purification.

### 2.2 Spectroscopic measurements

UV absorption spectra were recorded in a JASCO V-760 spectrophotometer. The Job’s plot analysis can be used to determine the stoichiometry of ligand@protein complexes by measuring the UV absorption spectra of mixtures containing different ligand@protein molar ratios that maintain the total molar concentration constant (Huang, 1982); in our case, eleven solutions with a total concentration of 20 μM in PBS were prepared varying the drug (or metabolite)@protein molar ratio: 100:0, 90:10, 80:20, 70:30, 60:40, 50:50, 40:60, 30:70, 20:80, 10:90, 0:100. Then, the maximum ligand absorbance multiplied by the corresponding HSA concentration was plotted against the corresponding protein mole fraction to obtain the binding stoichiometry of the complex from the maximum signal observed in the Job’s Plot.

Steady-state fluorescence spectra were recorded on an Edinburgh FS5 spectrofluorometer, provided with a monochromator in the wavelength range of 200–900 nm using an excitation wavelength of 340 nm at room temperature. Measurements on drug@protein complexes were performed in aerated PBS of 1:1 M ratio mixtures at 10 µM. The absorbance of the samples at the excitation wavelength was kept below 0.1. The fluorescence quantum yields were determined using anthracene in ethanol as reference (Montalti et al., 2006).

A modified Scatchard analysis has been used to determine the binding constants (*K*_*B*_) of GFT, GFT-M1 and GFT-M2 within HSA and HAG either from fluorescence (F) or absorption (A) measurements (Healy, 2007), following Eq. 1:

$$\frac{F_{\max}-F_{0}}{F-F_{0}}=\frac{1}{K_{B}}\cdot {\left[P\right]}^{-1}+1$$ (1)

where *F*_max_ is the fluorescence maximum when all possible ligand is bound to the protein, *F*_*0*_ is the fluorescence maximum of the free ligand, *F* is the fluorescence maximum observed for a given protein concentration *[P*], and *K*_*B*_ is the binding constant of the ligand with the protein. To calculate the *K*_*B*_ values by means of UV absorption spectroscopy, the same Eq. 1 has been applied but using *A* instead of *F*.

Time-resolved fluorescence measurements were performed with an EasyLife X system containing a sample compartment composed of an automated peltier cuvette holder to control the temperature at 24°C, a pulsed LED excitation source and a lifetime detector. The employed LED excitation source was 340 nm, with emission filter of WG370. The fluorescence lifetimes (τ_F_) were obtained upon fitting the decay traces by a non-linear fitting/deconvolution procedure *F(t) = Σa*_*i*_ *·*exp(*-t/τ*_*i*_) by means of a one- or two-exponential function, depending on the investigated system. All spectroscopic measurements were done in 10 × 10 mm^2^ quartz cuvettes at room temperature.

### 2.3 Molecular docking

These calculations were performed using GOLD program version 2020.3.0 (Jones et al., 1997), and the protein coordinates were taken from the crystal structures of HSA in complex with hemin and myristic acid (PDB ID 1O9X) (Zunszain et al., 2003) and of HAG in the unbound form (PDB ID 3KQ0) (Schönfeld et al., 2008). The experimental procedure was similar to that described for LAP, N-LAP and O-LAP in HSA (Andreu et al., 2020). For GFT and GFT-M2, the protonated forms of the morpholine moiety were employed since they predominate at physiological pH (Domotor et al., 2018).

### 2.4 Molecular dynamics simulation studies

The proteins in complex with the highest score solution obtained by docking were immersed in a truncated octahedron of TIP3P water molecules and neutralized using the molecular mechanics force field ff14SB and GAFF of AMBER (Case et al., 2021). The resulting systems were submitted to 100 ns of dynamic simulation following our previously reported protocol (Andreu et al., 2020). Briefly, the experimental procedure involved: (i) minimization and charge distribution of the ligands (GFT, GFT-M1 and GFT-M2) using Gaussian 09 (Frisch et al., 2009); (ii) generation and minimization of the binary GFT@protein, GFT-M1@protein and GFT-M2@protein complexes using the poses obtained by docking; and (iii) simulations of the resulting minimized ligand@protein complexes. The cpptraj module in AMBER 20 was used to analyze the trajectories and to calculate the root-mean-square deviation (rmsd) of the protein and the ligand during the simulation (Case et al., 2021). The molecular graphics program PyMOL (DeLano, 2008) was employed for visualization and depicting enzyme structures. For Figures related to HSA and HAG, the amino acid numbering described in PDB entries 1O9X and 3KQ0, respectively, was employed.

## 3 Results and discussion

As stated above, the photophysical properties of either GFT and GFT-M1 are strongly affected by the environment; hence, ICT states are detected in polar organic solvents, whereas LE species are predominantly formed in non-polar ones and in GFT@HSA; by contrast, ESPT is the main process occurring for GFT-M1 in protein medium (Tamarit et al., 2021; Tamarit et al., 2022). In view of this variability, the photobehavior of GFT-M2 was investigated here, first in organic solvents of different polarities to identify the excited species that can be formed upon irradiation with UVA light.

The UV absorption spectra of GFT-M2 were almost similar in all solvents (Supplementary Figure S1), while the fluorescence properties were strongly affected by the polarity (Supplementary Figure S2). By comparison with previous results on GFT and GFT-M1, emission from LE states was expected also for GFT-M2 in non-polar solvents. Actually, in these media, fluorescence maxima were found at λ_max_ *ca.* 390 nm with higher ϕ_F_ values and shorter τ_F_ than those detected in polar solvents, where intramolecular charge transfer (ICT) excited species are generally formed (Tamarit et al., 2021; Tamarit et al., 2022).

**FIGURE 2:**
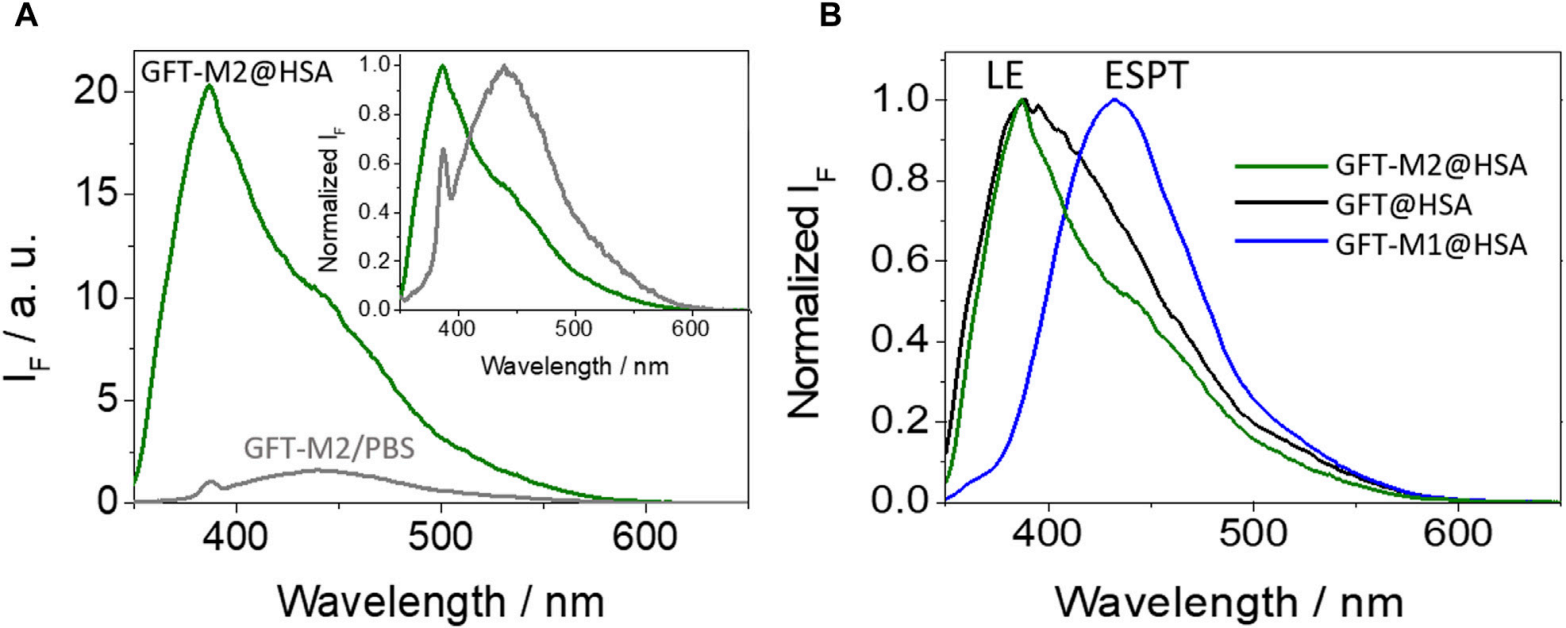
**(A)** Fluorescence spectra for GFT-M2 in PBS (gray) and for GFT-M2@HSA (green). The inset shows the normalized spectra. **(B)** Normalized fluorescence spectra for GFT-M2@HSA (green), GFT@HSA (black) and GFT-M1@HSA (blue). Measurements were performed at λ_exc_ = 340 nm in PBS; for ligand@HSA complexes, solutions were at 1:1 M ratio (10 μM).

The photobehavior of GFT-M2 in a more complex biological environment such as that provided by the HSA binding sites has been examined. First, Job’s plot analysis (Huang, 1982) allowed determination of a 1:1 stoichiometry for the protein-metabolite complex (Supplementary Figure S3). Its photophysical properties have been studied at λ_exc_ = 340 nm, where the protein does not absorb and GFT-M2 is selectively irradiated (Supplementary Figure S4). As it can be observed from Figure 2A, a noticeable fluorescence enhancement was observed upon binding with the protein. Interestingly, the emission profile of GFT-M2 strongly varied depending on the microenvironment; so, a maximum at *ca.* 440 nm was detected for GFT-M2 free in the bulk solution, while a shift towards much shorter wavelengths (∼388 nm) occurred upon binding with HSA (inset in Figure 2A). Since GFT-M2 is a phenol, this can be explained as a result of a proton transfer process occurring in the bulk solution, which is hindered within the protein cavities; this is supported by UV absorption spectroscopy, where the shoulder between 370 and 400 nm (Supplementary Figure S4C), associated to phenolates, practically disappears in the supramolecular complex. This effect is even clearer at different GFT-M2/HSA ratios, where formation of phenolate-like species was lower at high protein concentrations (Supplementary Figure S5A). These results contrast with those previously observed for GFT-M1 (Supplementary Figures S4B, S5B), which is also a phenol but ESPT is the main process within HSA thanks to hydrogen bonding with Val116 (Tamarit et al., 2022). Therefore, the emission profile of GFT-M2 within HSA is comparable with that of the protein-bound GFT (Figure 2B); consequently, LE states are mainly formed in GFT-M2@HSA.

**FIGURE 3:**
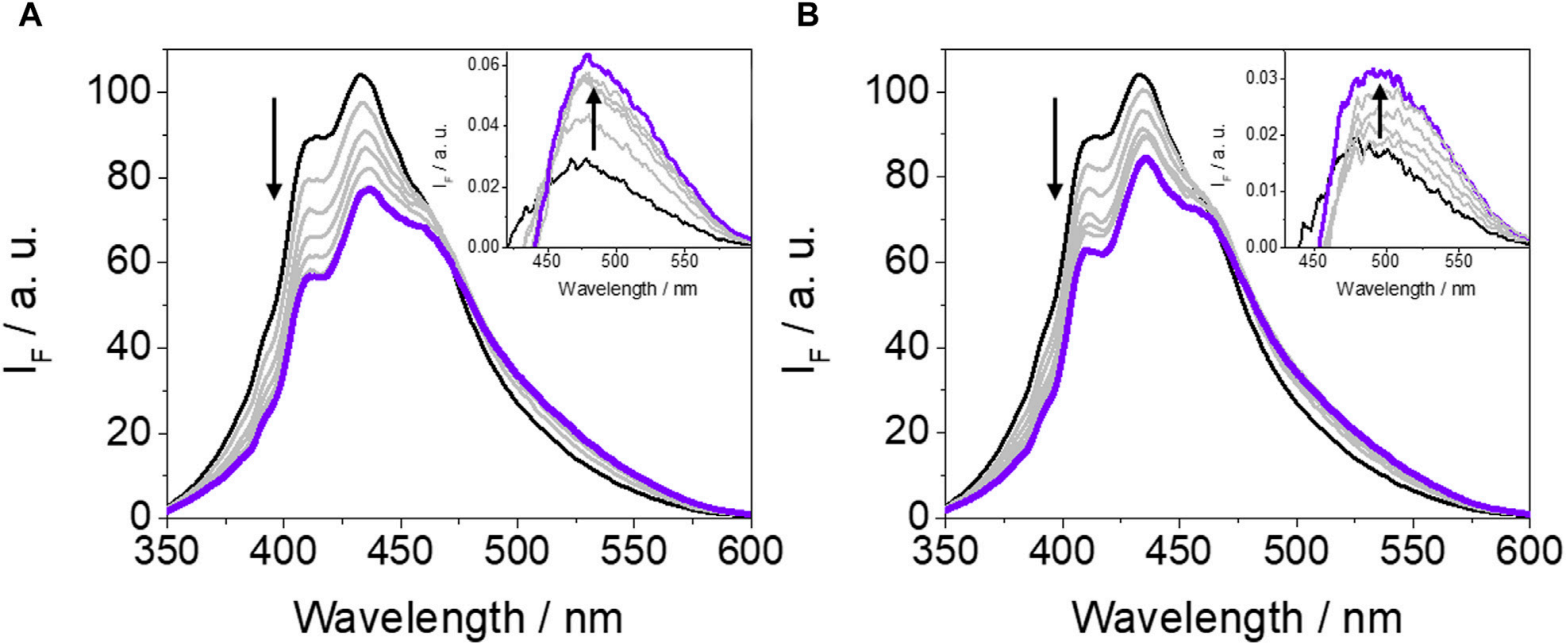
Fluorescence spectra of GFT-M2 after addition of increasing amounts of Tyr **(A)** or Trp **(B)**. All measurements were performed at metabolite:amino acid molar ratios of 1:50, 1:100, 1:150, 1:200, 1:250 and 1:300 after excitation at 340 nm in toluene. The concentration of GFT-M2 was maintained constant at 10 μM. The insets show the formation of a new band, which is associated with formation of phenolate-like species through interaction between hydrogen-bonded GFT-M2 and Tyr or Trp in the ground state; these bands were determined upon subtraction of the emission from excited GFT-M2 to those of GFT-M2/amino acid (Tyr or Trp) at the different molar ratios.

An important point to discuss is the low fluorescence yield value of GFT-M2 within the protein cavities (ϕ_F_ ∼ 0.01) compared with that obtained in cyclohexane (ϕ_F_ ∼ 0.13). This effect has been previously observed for GFT, where ϕ_F_ decreases from ∼0.19 in cyclohexane to ∼0.02 within HSA, and it is associated with quenching of LE states through photoinduced electron transfer from Tyr and/or Trp. It is worth to mention that this is a dynamic process that lowers τ_F_ values (Tamarit et al., 2021; Tamarit et al., 2022). With the aim of investigating this possibility for GFT-M2, fluorescence measurements were performed in the non-polar solvent toluene in the presence of increasing amounts of Tyr and Trp (due to solubility requirements, the *N*-acetyl methyl ester amino acid derivatives, namely, NAc-TyrMe or NAc-TrpMe, were used). Interestingly, fluorescence quenching of the LE singlet state of the metabolite (^1^GFT-M2*) was indeed observed upon addition of Tyr or Trp (Figure 3); however, the process was found to be static in nature since the fluorescence lifetimes were hardly affected (the lifetime of ^1^GFT-M2* in toluene was constant at *ca*. 2.42 ns at the different GFT-M2/amino acid molar ratios: 1:50, 1:100, 1:150, 1:200, 1:250 and 1:300). It is worth to mention that using either Tyr or Trp as quenchers, a new band with λ_max_ ∼ 490 nm arose at high amino acid concentrations (insets of Figure 3); this is attributed to emission from phenolate-like species involving interaction between hydrogen-bonded GFT-M2 and Tyr (or Trp) in the ground state. However, and as discussed above, these species have not been observed in the supramolecular GFT-M2@HSA complex, since LE states predominate. Therefore, it would exist an additional deactivation route for ^1^GFT-M2* within the protein, which would be strongly affected by the conformational arrangement of the metabolite in the binding sites (see the discussion on MD simulations).

An additional point to address is the strength of interaction of the drug and its two metabolites within HSA, since it is key for their transport to the specific targets and for their toxicological activity. Although there are many analytical techniques to determine the binding constant (*K*_*B*_) between a drug and a protein, fluorescence spectroscopy is a widely used one due to its high sensitivity and selectivity (Hirose, 2001; Vuignier et al., 2010; Vayá et al., 2014; Dos Santos Rodrigues et al., 2023). In this context, there are different *K*_*B*_ values reported for GFT within HSA, which vary in the range of 10^3^–2×10^5^ M^-1^ (Li et al., 2006; Kabir et al., 2016; Wu et al., 2016; Domotor et al., 2018; Tanzadehpanah et al., 2018; Tanzadehpanah et al., 2019). In view of this discrepancy, up to two-orders of magnitude, we determined the binding constants for the drug and their two metabolites within HSA by means of UV absorption and fluorescence spectroscopies. To this end, a modified Scatchard analysis was performed (Supplementary Figures S6–S8). (Healy, 2007) The *K*_*B*_ values obtained by the two techniques were very similar, with deviations between them lower than 10%; the obtained data are summarized in Table 1. As it can be observed, the strength of interaction of the two metabolites to HSA is slightly higher than that determined for the parent drug.

**TABLE 1:** Binding constants for GFT, GFT-M1 and GFT-M2 within HSA and HAG.

*K*_*B*_/M^-1^	HSA	HAG
GFT	7.5 ± 0.24 × 10^4^	1.2 ± 0.08 × 10^5^
GFT-M1	1.2 ± 0.06 × 10^5^	9.1 ± 0.32 × 10^4^
GFT-M2	1.8 ± 0.16 × 10^5^	1.5 ± 0.05 × 10^5^

**FIGURE 4:**
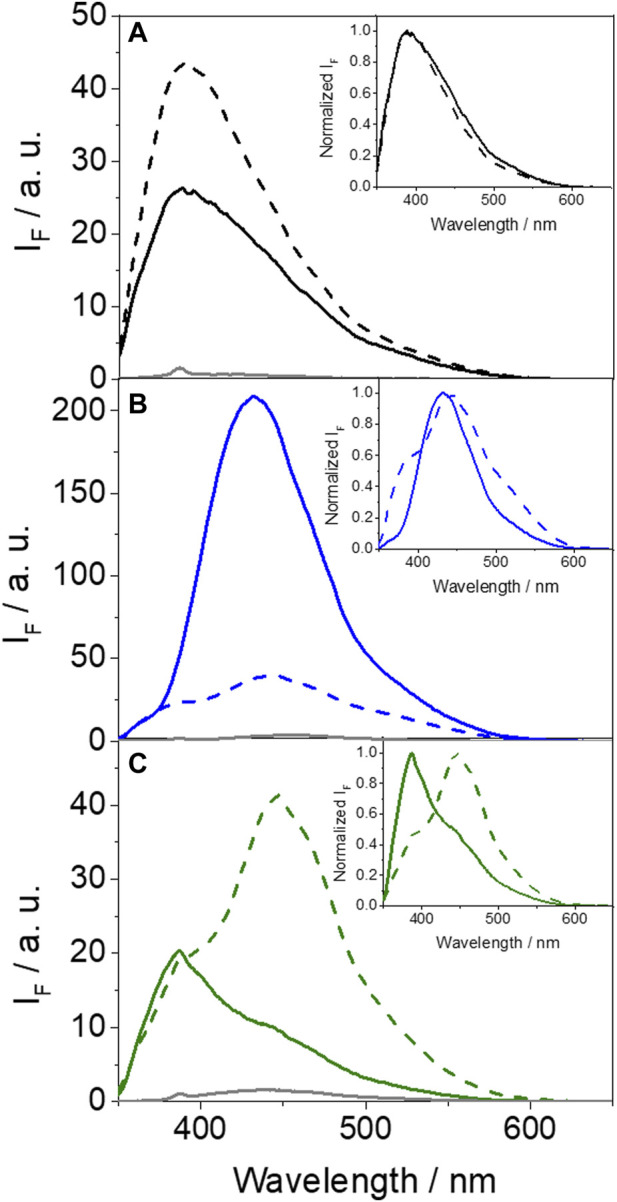
Fluorescence spectra at λ_exc_ = 340 nm in PBS for **(A)** GFT (gray), GFT@HSA (solid black) and GFT@HAG (dashed black), **(B)** GFT-M1 (gray), GFT-M1@HSA (solid blue) and GFT-M1@HAG (dashed blue), and **(C)** GFT-M2 (gray), GFT-M2@HSA (solid green) and GFT-M2@HAG (dashed green). The insets show the normalized spectra. For ligand@protein complexes, solutions were at 1:1 M ratio (10 μM).

A similar study was performed for HAG, since it is another important transport protein in human plasma. In this context, fluorescence experiments were also performed at λ_exc_ = 340 nm, were the drug and/or its two metabolites are the only absorbing species (Supplementary Figure S9). As expected, a noticeable fluorescence enhancement for either GFT, GFT-M1 and GFT-M2 was observed upon binding with HAG (Figure 4). Job’s plot analyses confirmed that 1:1 stoichiometry complexes are formed between the drug or its metabolites and HAG (Supplementary Figure S10).

**FIGURE 5:**
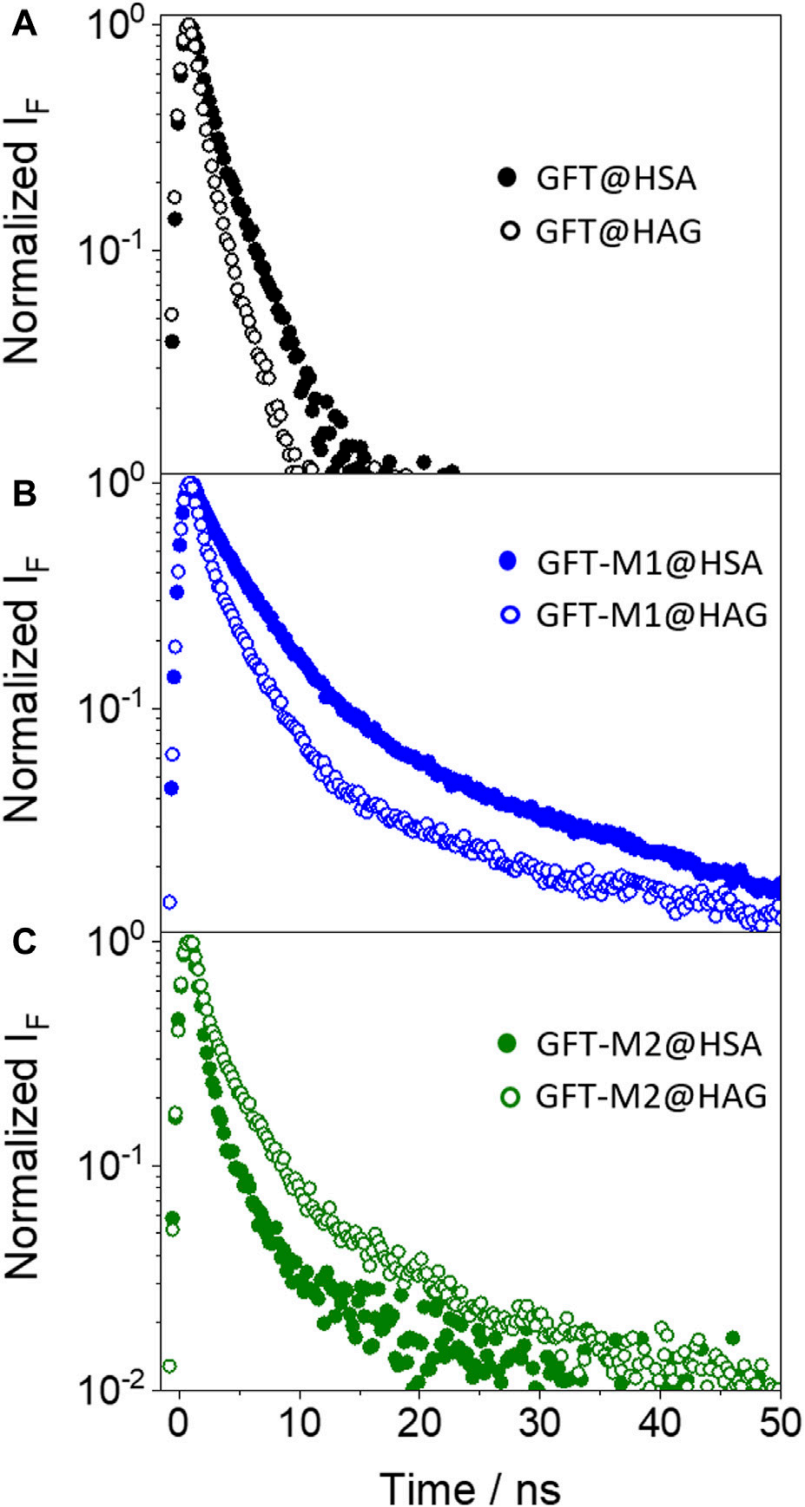
Fluorescence decays for **(A)** GFT@HSA (solid black circles) and GFT@HAG (opened black circles), **(B)** GFT-M1@HSA (solid blue circles) and GFT-M1@HAG (opened blue circles), and **(C)** GFT-M2@HSA (solid green circles) and GFT-M2@HAG (opened green circles). Measurements were performed at λ_exc_ = 340 nm for ligand@protein solutions at 1:1 M ratio (10 μM) in PBS. The fluorescence lifetimes were obtained upon fitting the decay traces by a non-linear fitting/deconvolution procedure *F(t) = Σa*_*i*_·exp(*-t/τ*_*i*_) by means of a one- or two-exponential function, depending on the investigated system. In general, for all decay fits, the adjusted *R* ^2^ value ranged from 0.996 to 0.998.

In the case of GFT, its fluorescence profile within HAG was very similar to that observed for HSA (Tamarit et al., 2021), showing a maximum centered at *ca.* 390 nm and displaying slightly higher ϕ_F_ (Figure 4A; Table 2). Accordingly, emission is again associated with LE states as the only excited species; this was further supported by time-resolved fluorescence measurements, since a one-exponential law was used to get a good fitting of the kinetic traces (Figure 5A). Likewise, comparable results were obtained for GFT-M1 bound to HSA and HAG; the fluorescence bands peak at *ca.* 432 and 442 nm, respectively (Figure 4B). This is in line with emission from phenolate-like excited states, which display longer lifetimes than LE (Figure 5B; Table 2). Interestingly, a shoulder at around 390 nm was detected for GFT-M1@HAG, which might be associated with emission from LE states to a lower extent than phenolate-like species. In fact, a two-exponential function was necessary to get a good fitting for the fluorescence kinetics, where the short component (∼0.5 ns) is associated to emission from LE states while the longer one (∼3.1 ns) is assigned to phenolate species (Figure 5B). A completely different photobehavior was observed for the protein-bound GFT-M2. As explained above, emission from LE states predominates in HSA, whereas phenolate-like species are mainly formed in HAG (λ_max_ ∼ 446 nm), although LE states also appears to a much lower extent, since a shoulder at ∼ 388 nm was also detected. These results are again supported by time-resolved fluorescence measurements (Figure 5C), where a longer-lived component assigned to phenolate-like species dominates the kinetics of GFT-M2@HAG.

**TABLE 2:** Fluorescence properties of the protein-bound drug (or metabolite) complexes at λ_exc_ = 340 nm in PBS.

	HSA			HAG		
	λ_max_/nm	ϕ_F_	τ_F_/ns	λ_max_/nm	ϕ_F_	τ_F_/ns
GFT	390^a^	0.02^a^	1.3^a^	390	0.03	0.8
GFT-M1	432^b^	0.15^b^	2.5^b^	442 (390)^c^	0.04	1.6^d^
						*τ*_*1*_ *0.5* (*28%*)
						*τ*_*2*_ *3.1* (*72%*)
GFT-M2	388	0.01	1.0^d^	446 (388)^c^	0.03	1.7^d^
			*τ*_*1*_ *0.6* (*75%*)			*τ*_*1*_ *0.7* (*35%*)
			*τ*_*2*_ *4.9* (*25%*)			*τ*_*2*_ *4.1* (*65%*)

^a^ Data from ref. 35.

^b^ Data from ref. 36.

^c^ Shoulders detected in the fluorescence spectra are shown between brackets. A one-exponential function was used to fit all the decay traces, except for *d*, where a mean lifetime was determined as <τ_F_> = a_1_τ_1_ + a_2_τ_2_; the τ_1_ and τ_2_ values, in addition with the weight of each component, are also shown.

**FIGURE 6:**
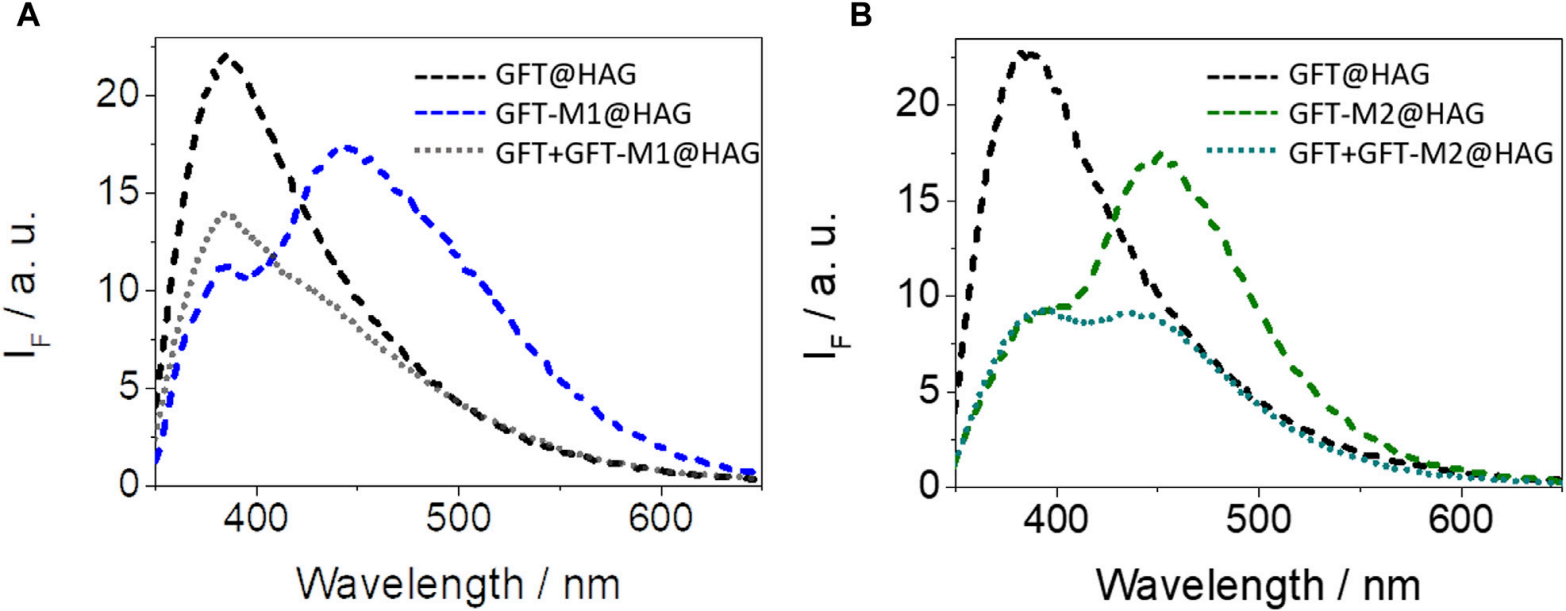
Fluorescence spectra at λ_exc_ = 340 nm for **(A)** GFT@HAG (dashed black), GFT-M1@HAG (dashed blue) and GFT + GFT-M1@HAG (dotted gray), and **(B)** GFT@HAG (dashed black), GFT-M2@HAG (dashed green) and GFT + GFT-M2@HAG (dotted dark green). All mixtures were at 1:1 M ratio (10 µM) in PBS. For drug + metabolite/HAG, equimolar 1:1:1 mixtures were used.

The strength of interactions of GFT and its two metabolites within HAG was also studied by means of spectroscopic techniques (Supplementary Figures S11–S13). The *K*_*B*_ values (Table 1) are on the order of those obtained for HSA, and in the case of GFT, agree well with the reported data (Li et al., 2006). Competing interactions on mixtures containing the drug and a metabolite in the presence of HAG confirmed the consistency of the determined binding constants. Hence, the emission profile of GFT + GFT-M1 in the presence of HAG resembles that of GFT@HAG, in agreement with its higher *K*_*B*_ value (Figure 6A). By contrast, the fluorescence spectrum for GFT + GFT-M2 in the presence of HAG might contain equal contributions from both GFT@HAG and GFT-M2@HAG (Figure 6B), which agrees with the similar strength of interactions of the two supramolecular complexes (1.2 × 10^5^ and 1.5 × 10^5^ M^-1^, respectively).

Moreover, competing interactions of the drug (or its metabolites) in a mixture containing the two proteins are also in line with the *K*_*B*_ values obtained experimentally. In this context, the emission of GFT in the presence of an equimolar mixture of the two proteins resembles that of GFT@HAG (Supplementary Figure S14A), confirming its higher binding constant value. A similar conclusion can be drawn for GFT-M1 and GFT-M2 in the presence of an equimolar mixture of HSA and HAG, where higher affinity to HSA was observed for the former, while similar affinity to either HSA or HAG was noticed for GFT-M2 (Supplementary Figures S14B, C, respectively).

The photobehavior herein discussed can justify the differences in the photosensitivity disorders previously observed by excitation of GFT, GFT-M1 and GFT-M2 in a biological media with UVA light (El Ouardi et al., 2023). In this regard, GFT-M1 displays the highest fluorescence yield in the protein environment, in particular in the presence of HSA. Besides, the fluorescence lifetime observed within this protein was the longest one. This can explain the highest phototoxicity detected for GFT-M1. Accordingly, GFT is also phototoxic but to a lesser extent, in line with its lower ϕ_F_ value and shorter τ_F_, while GFT-M2 is much less phototoxic (El Ouardi et al., 2023). Interestingly, since electron and proton transfer processes are expected to occur in the protein environment, the photosensitizing damage from either the drug and its two metabolites is consistent with the involvement of a type I mechanism. Managing photosensitivity reactions involves diverse strategies, including medication adjustment, symptom monitoring and photoprotection. Since each patient can metabolize drugs yielding a personalized profile of metabolites, in the case of enhanced GFT-M1 production it might be appropriate to reduce the administered drug doses as well as avoiding Sun exposure and/or prescribing UVB plus UVA photoprotection. As a precaution, these measures would be advisable for all patients receiving GFT medication.

To provide a more in-depth understanding of the molecular bases responsible for the distinct photobehavior of the GFT metabolites, GFT-M1 and GFT-M2, relay on the protein that transport them, their binding modes were studied *in silico*. To this end, docking studies were first performed using the GOLD program version 2021.3.0 (Jones et al., 1997), followed by MD simulation studies to provide a more realistic picture of the ligand arrangement upon binding. The protein coordinates of the reported wild-type structure of HAG (PDB ID 3KQ0) and of HSA in complex with myristic acid and hemin (PDB ID 1O9X) were used for these studies (Zunszain et al., 2003; Schönfeld et al., 2008). The most plausible ligand@protein complexes obtained by docking were immersed in a truncated octahedron box of TIP3P water molecules and neutralized by addition of sodium ions, and then subjected to 100 ns of dynamic simulation using the molecular mechanics force field AMBER ff14SB and GAFF (Case et al., 2021).

**FIGURE 7:**
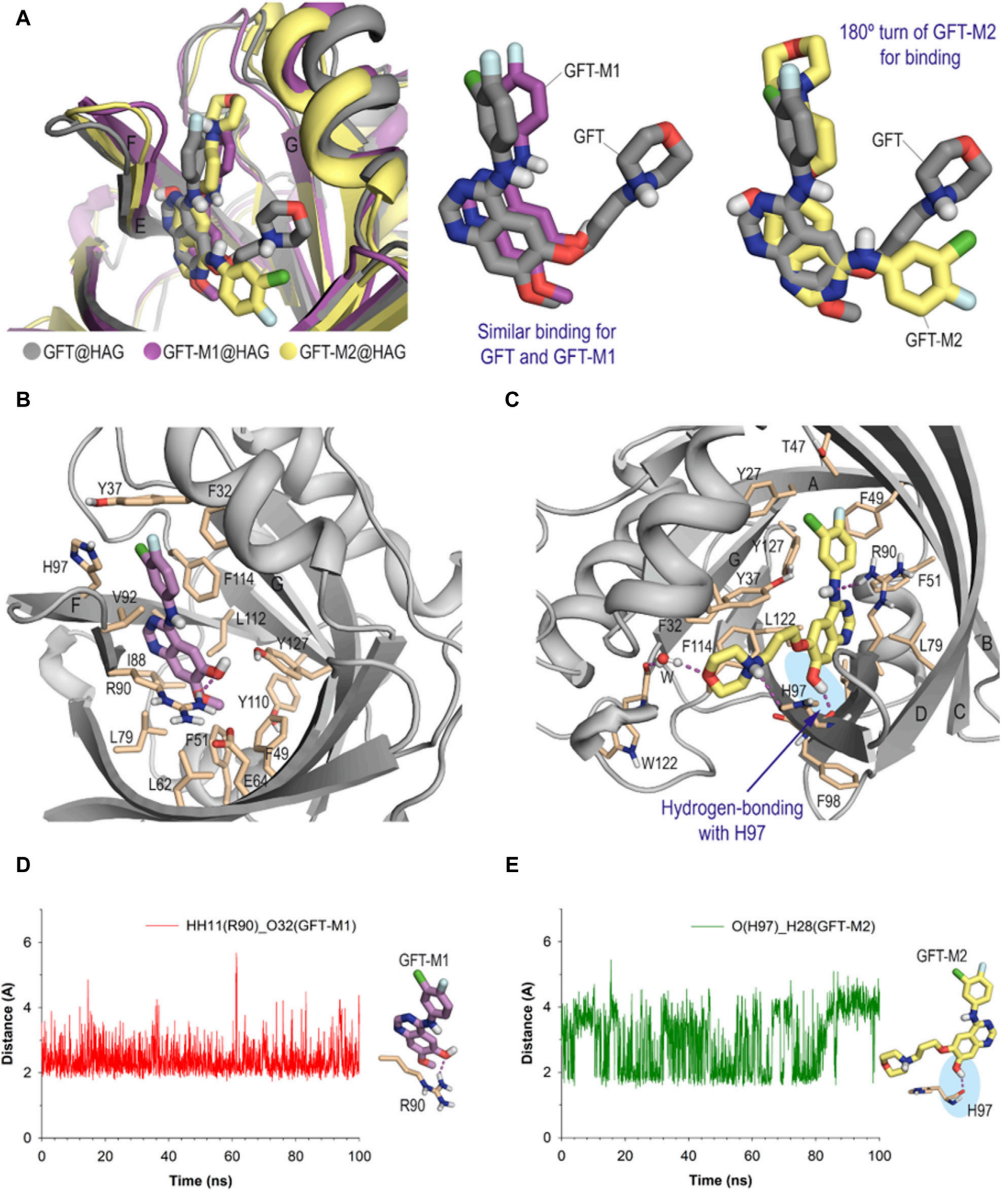
Binding mode of GFT and its metabolites with HAG obtained by MD simulation studies. **(A)** Comparison of the GFT@HAG (gray), GFT-M1@HAG (magenta) and GFT-M2@HAG (yellow) binary complexes (snapshots after 90, 90 and 80 ns, respectively, are shown) and superposition of the arrangements of GFT and GFT-M1 and GFT and GFT-M2, which are shown in sticks. Note how while GFT and GFT-M1 show a similar arrangement, GTF-M2 undergoes a 180° turn for binding. **(B, C)** Detailed views of GFT-M1 **(B)** and GFT-M2 **(C)** binding modes. Snapshots after 90 ns and 80 ns, respectively, are shown. **(C)** Detailed view of GFT-M2 binding mode. Snapshot after 80 ns is shown. Hydrogen-bonding interactions between the ligand and protein residues are shown as dashed lines (magenta). Note how the phenol moiety interacts by hydrogen-bonding with the main carbonyl group of H97 (blue shadow). A similar interaction is not observed for GFT-M1. **(D, E)** Variation of the relative distances between: **(D)** the phenol moiety (O atom) in GFT-M1 and the guanidinium group of R90 (HH11 atom) in the GFT-M1@HAG protein complex; **(E)** the phenol moiety (H atom) in GFT-M2 and the main carbonyl group of H97 (O atom) in the GFT-M2@HAG protein complex, during whole simulation. Average values of 2.9 Å and 2.4 Å, respectively.

As stated above, HAG contains a single large and flexible cavity to bind drugs (Kremer et al., 1988; Maruyama et al., 1990). The *in silico* results showed that GFT and its metabolites would be stable within the corresponding protein recognition sites, as revealed by the low rmsd (root-mean-square deviation) values obtained for the protein backbone and ligands during the whole simulation (Supplementary Figures S15, S16). More importantly, the two metabolites showed markedly different binding behavior depending on the transport protein used when compared with the parent drug. Thus, the interaction of GFT-M2 with HAG has been found to be much different than those of GFT and GFT-M1, which would be quite similar among them (Figure 7A). For GFT and GFT-M1, the pyrimidine ring of the quinazoline core would be located close to β-sheets F and E, and the 3-chloro-4-fluorophenyl moiety would be placed between β-sheets F and G (Figure 7B). On the contrary, GFT-M2 would undergo a 180° turn for binding, thus placing the phenyl group pointing towards β-sheets A and B and the morpholinyl moiety between β-sheets F and G (Figure 7C). Under the latter arrangement, the phenolic proton in GFT-M2 would establish a hydrogen-bonding interaction with the main carbonyl group of residue H97, which showed to be stable during most of the simulation (Figure 7E). It was also observed that when this interaction is lost as result of the rotation of OH group, a similar hydrogen-bonding interaction with the main carbonyl group of residue F98 would be established. For GFT-M1, the interaction of one of the oxygen lone pair of its OH group with the guanidinium group of R90 (average value of 2.4 Å during 100 ns-simulation) would freeze the orientation of the phenolic proton towards the bulky water solvent (Figure 7D). As a result, no interactions by hydrogen-bonding between its phenolic proton and any residue of the protein were identified.

**FIGURE 8:**
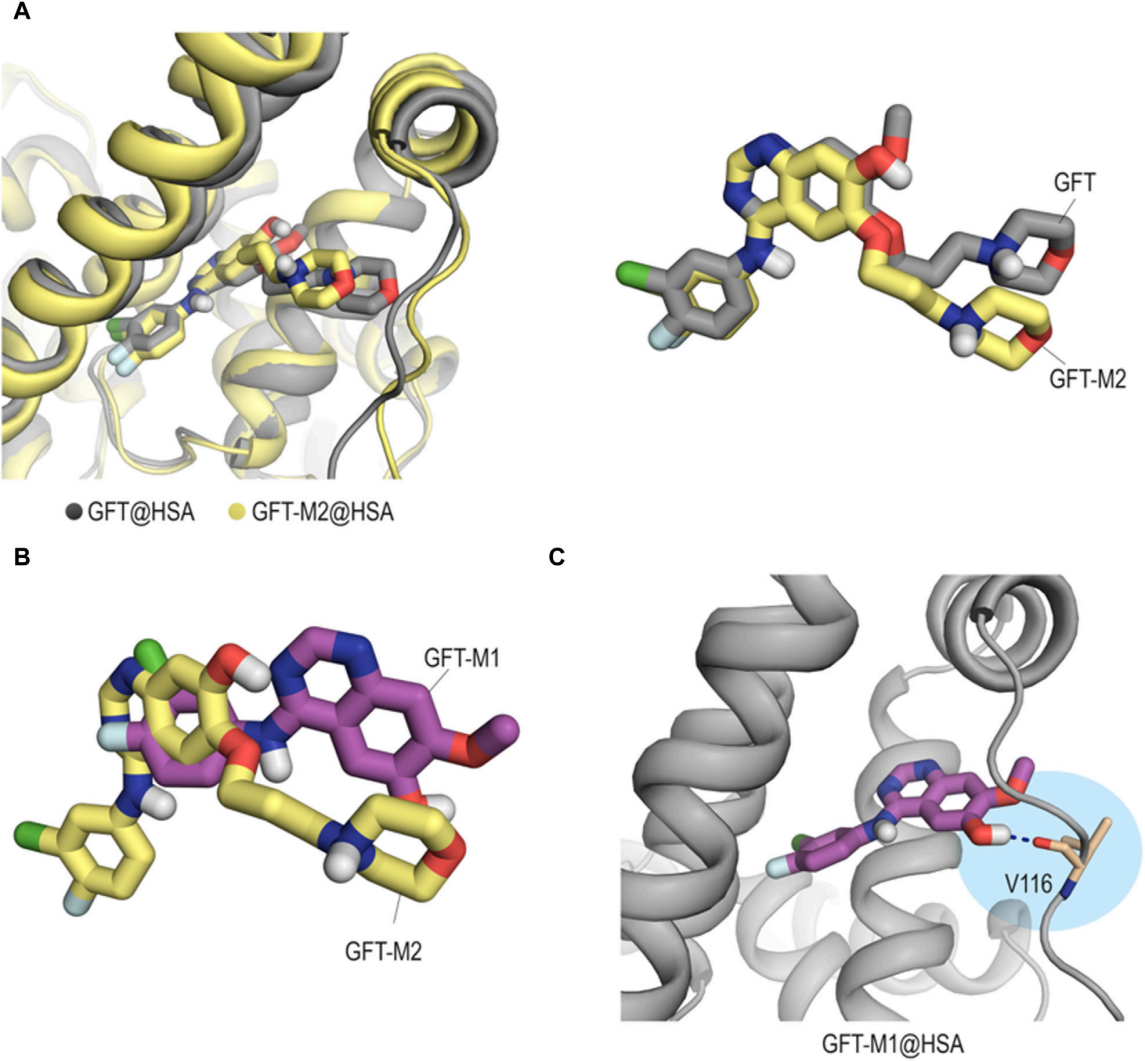
Binding mode of GFT and its metabolites with HSA obtained by MD simulation studies. **(A)** Comparison of the binary GFT@HSA (gray) and GFT-M2@HSA (yellow) complexes and overlapping of the arrangements of GFT and GFT-M2, which are shown in sticks. Snapshots after 100 ns are shown. **(B)** Superposition of the arrangements of GFT-M1 (magenta) and GFT-M2 (yellow). **(C)** Close view of GFT-M1 binding mode with HSA. Snapshot taken after 90 ns of simulation. Hydrogen-bonding interactions are shown as blue dashed lines. Contrary to what happens with GFT-M2, the phenolic proton in GFT-M1 establishes a hydrogen-bonding interaction with the protein (residue V116).

Concerning HSA, it contains the major cavities (site I, II and III) where drugs can interact (Sudlow et al., 1976; Zsila, 2013). Previous reports show that GFT binds to site III (Tamarit et al., 2021), so that it was selected for these studies. Our computational studies revealed that unlike what happens with HAG, the interactions of GFT and its metabolites with site III (subdomain IB) of HSA does not follow the previous pattern. Thus, while GFT and GFT-M2 would achieve a similar arrangement (Figure 8A), GFT-M1 would be buried at the bottom of the pocket thanks to the lack of the morpholine moiety (Figure 8B). More importantly, this insertion into the cleft would be promoted by a hydrogen-bonding interaction between the phenolic proton in GFT-M1 and the main carbonyl group in residue V116 (Figure 8C). For GFT-M2, a similar interaction was not identified during the whole simulation.

## 4 Conclusion

The photobehavior of the anticancer drug GFT and its two photoactive metabolites GFT-M1 and GFT-M2 has been investigated in the presence of the most abundant transport proteins in human plasma, *i.e.,* HSA and HAG. The strength of binding and the nature of the main transient species that are formed upon irradiation of the protein-bound drug (or metabolite) with UVA have been studied by means of spectroscopic techniques. In general, the protein strongly modulates the fate of the excited species that are formed in the confined biological environment. In this regard, excitation of GFT-M2@HSA at 340 nm leads mainly to formation of locally excited states, whereas phenolate-like species predominate in HAG. By contrast, a diverging behavior is observed for GFT-M1, which forms phenolate-like species as the only excited states in HSA, while locally excited states are also formed within HAG. As regards GFT, locally excited states are primarily formed in the two proteins. These results are supported by molecular dynamics simulations, which rationalize the variability detected in the photoinduced processes and the type of excited species formed in the protein cavities based on the differences in the ligand binding mode, the type of interactions with the protein, and the arrangement of key functional groups involved in the ligand structure. Taken together, the herein reported studies highlight the relevant role of the biomacromolecule microenvironment in the modulation of the photobiological properties of the ligand inherent to its chemical structure.

## Data Availability

The original contributions presented in the study are included in the article/Supplementary Material, further inquiries can be directed to the corresponding authors.
