# Supplementary material for: Modulation of the photobehavior of gefitinib and its phenolic metabolites by human transport proteins
Source: Front Pharmacol. 2024 May 16;15:1387057. doi: 10.3389/fphar.2024.1387057 (PMC11137198; doi:10.3389/fphar.2024.1387057)
Supplement: Supplementary file 1 [file DataSheet1.pdf]

Supplementary Material

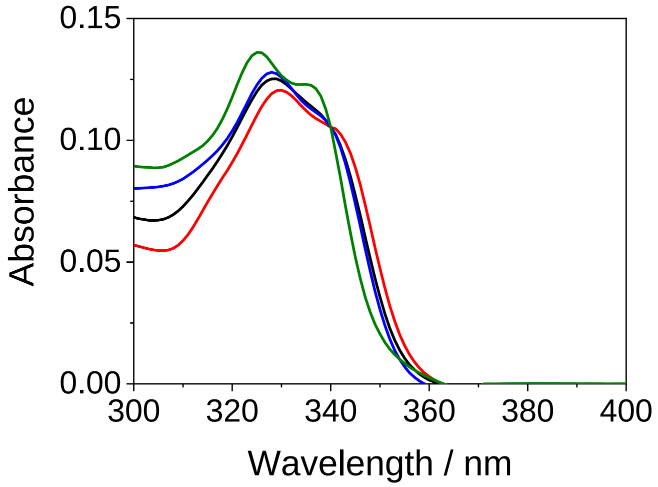

FIGURE S1. UV absorption spectra of 10 μM solutions for GFT-M2 in acetonitrile (black), 1,4-dioxane (red), toluene (blue) and cyclohexane (green).

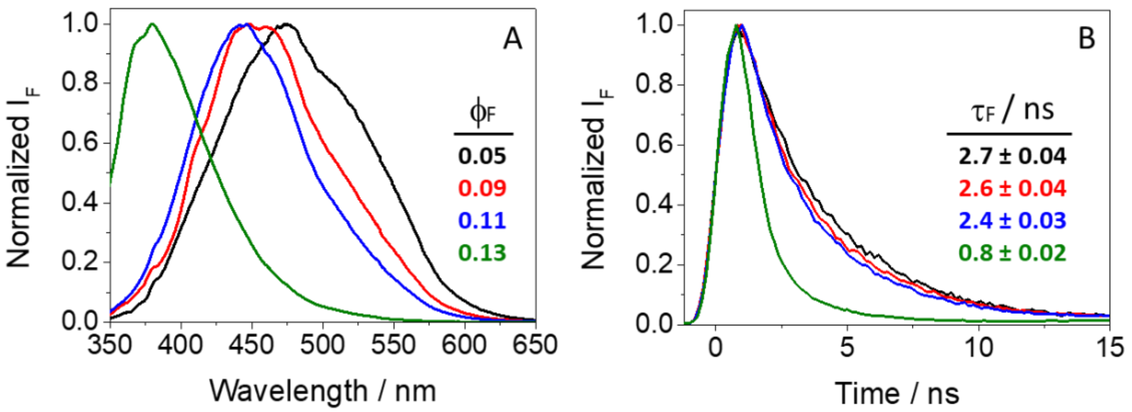

FIGURE S2. Normalized fluorescence spectra (A) and decay traces (B) for GFT-M2 in acetonitrile (black), 1,4-dioxane (red), toluene (blue) and cyclohexane (green) after excitation at 340 nm. The fluorescence quantum yields (ϕ_F) and lifetimes (τ_F) are also shown. For all decay fits, the adjusted R^2 value was *ca*. 0.998.

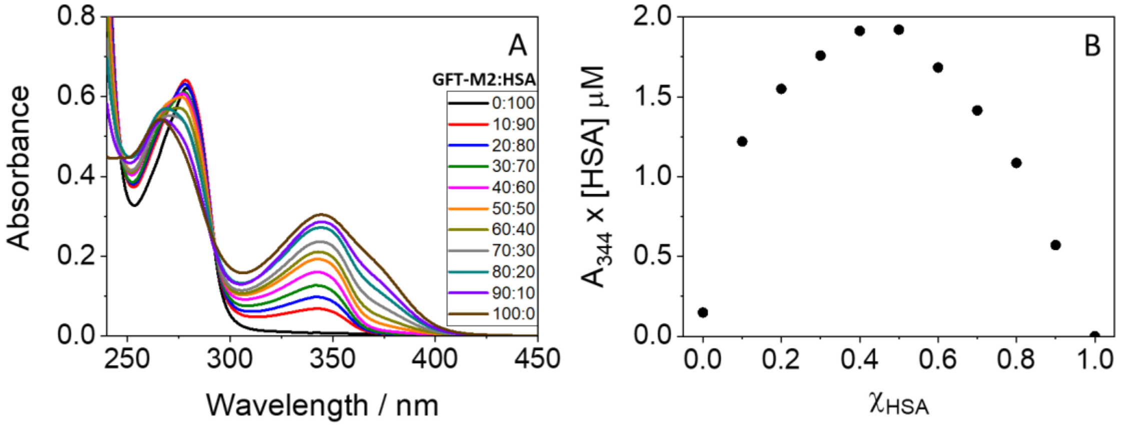

FIGURE S3. UV absorption spectra (A) and UV absorption Job's plot (B) for GFT-M2@HSA at a total concentration of 10 μM in PBS.

The table shows the values used for the Job's plot analysis.

χ_{HSA}	[HSA] (μM)	A344	[HSA]×A344
**0**	20	0.00744	0.14876
**0.1**	18	0.06778	1.22012
**0.2**	16	0.09688	1.55016
**0.3**	14	0.12562	1.75872
**0.4**	12	0.15943	1.91311
**0.5**	10	0.19204	1.92042
**0.6**	8	0.2104	1.6832
**0.7**	6	0.23591	1.41548
**0.8**	4	0.27149	1.08596
**0.9**	2	0.28571	0.57142
**1**	0	0.30386	0

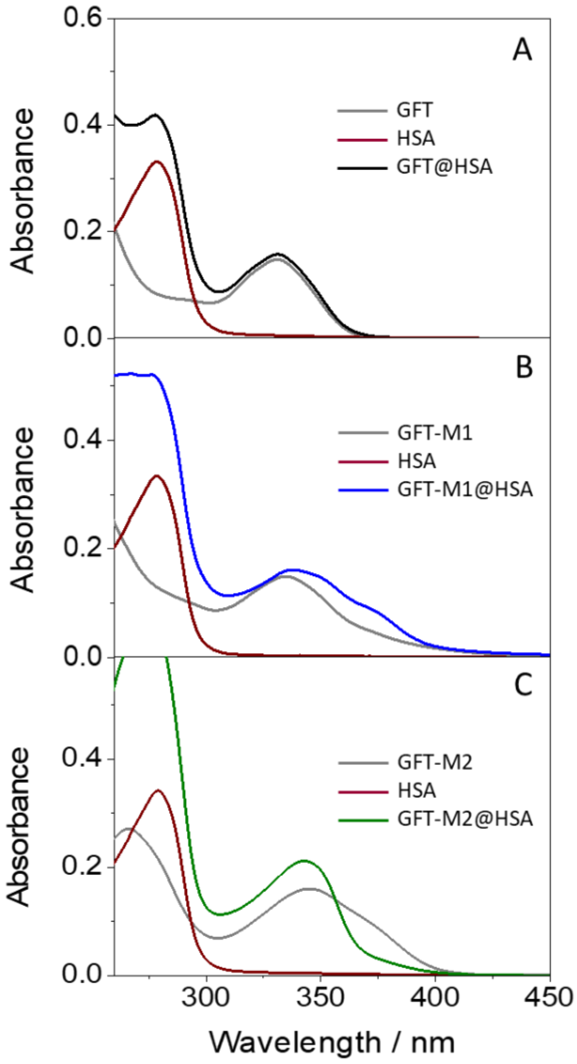

FIGURE S4. UV absorption spectra for (A) HSA (dark red), GFT (gray) and GFT@HSA (black), (B) HSA (dark red), GFT-M1 (gray) and GFT-M1@HSA (blue), and (C) HSA (dark red), GFT-M2 (gray) and GFT-M2@HSA (green). All measurements were performed at 10 µM in PBS. For ligand@HSA complexes, solutions were at 1:1 molar ratio.

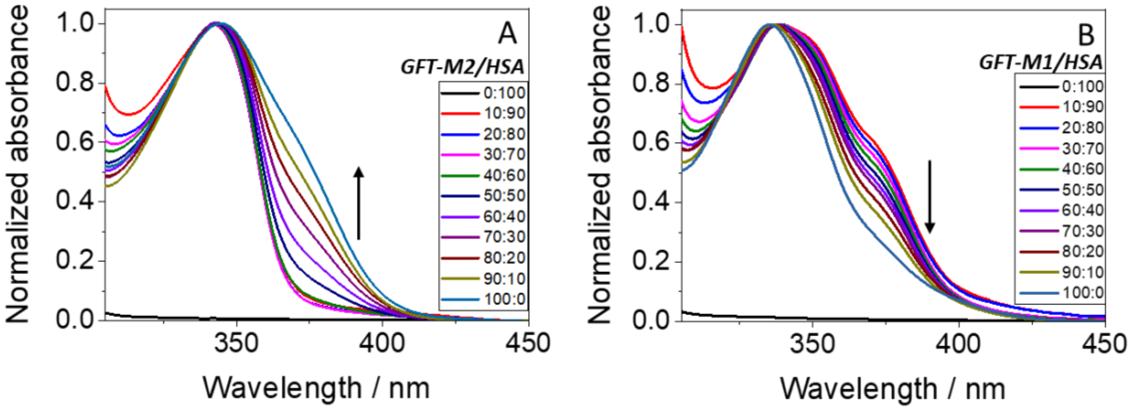

FIGURE S5. Normalized UV spectra for mixtures containing GFT-M2/HSA (A) and GFT-M1/HSA (B) in aqueous PBS.

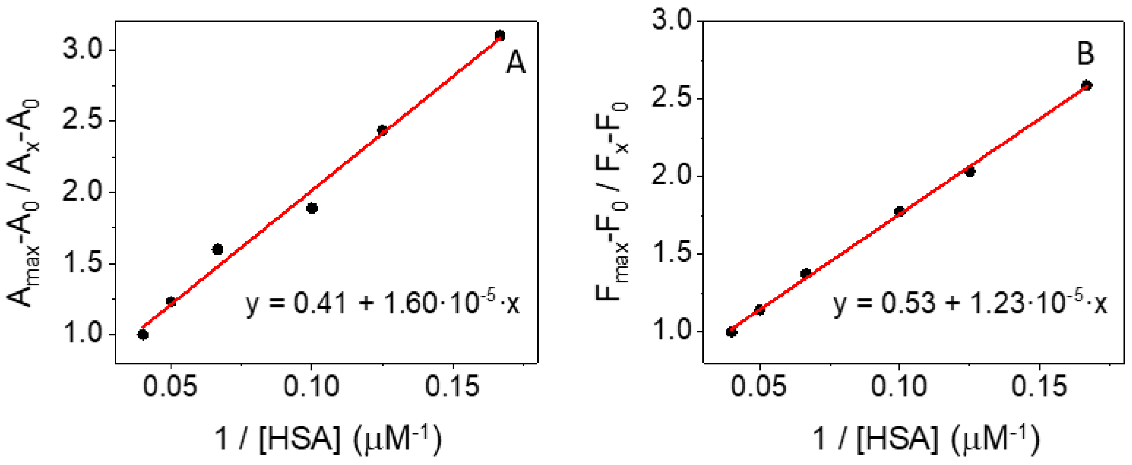

FIGURE S6. Linear fit of the modified Scatchard plot analysis to determine the K_B values for GFT@HSA by means of UV absorption (A) or fluorescence (B) spectroscopies.

The tables show the data used to determine the K_B values for GFT@HSA.

GFT@HSA (A0 = 0.12488)

1/[HSA]	A (331 nm)	Amax-A0/Ax-A0
166666.66667	0.13037	3.10269
125000	0.13187	2.43672
100000	0.1339	1.88893
66489.3617	0.13553	1.59925
50000	0.1387	1.23291
39936.10224	0.14192	0.99977

GFT@HSA (IF0 = 1415.11047)

1/[HSA]	IF (390 nm)	IFmax-IF0/IFx-IF0
166666.66667	91932.1406	2.59044
125000	116642.234	2.03493
100000	133319.781	1.77764
66489.3617	171803.547	1.37615
50000	206344.922	1.14419
39936.10224	235894.391	1

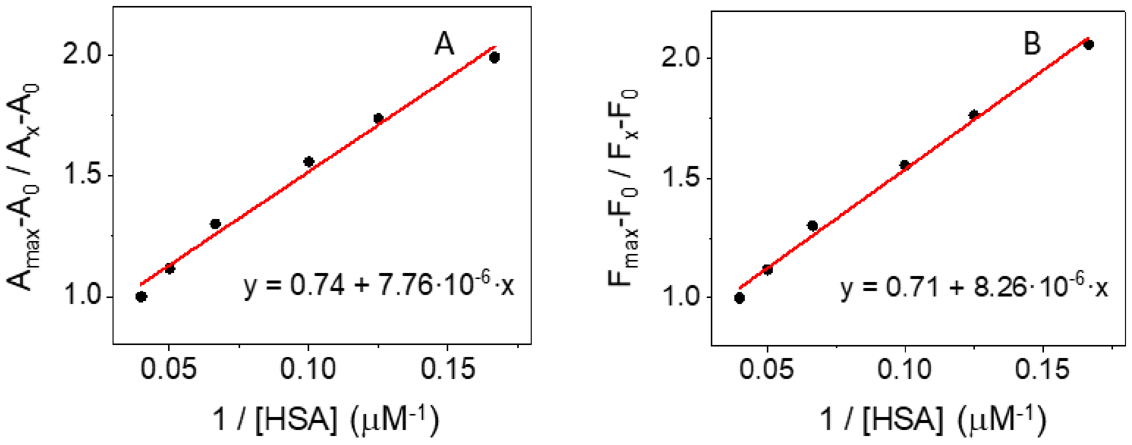

FIGURE S7. Linear fit of the modified Scatchard plot analysis to determine the K_B values for GFT-M1@HSA by means of UV absorption (A) or fluorescence (B) spectroscopies.

The tables show the data used to determine the K_B values for GFT-M1@HSA.

GFT-M1@HSA (A0 = 0.08406)

1/[HSA]	A (335 nm)	Amax-A0/Ax-A0
166666.66667	0.09557	1.99082
125000	0.09667	1.73714
100000	0.09721	1.55889
66489.3617	0.09832	1.30151
50000	0.10094	1.11662
39936.10224	0.10369	1.00004

GFT-M1@HSA (IF0 = 6030.37988)

1/[HSA]	IF (431 nm)	IFmax-IF0/IFx-IF0
166666.66667	333147.156	2.05988
125000	387897.5	1.76454
100000	439802.188	1.5534
66489.3617	523271.594	1.30272
50000	608524.563	1.11839
39936.10224	679851.25	1

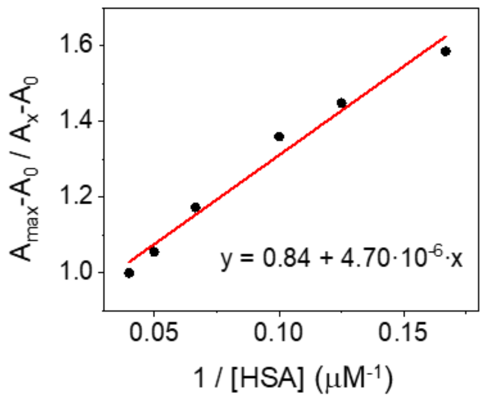

FIGURE S8. Linear fit of the modified Scatchard plot analysis to determine the K_B value for GFT-M2@HSA by means of UV absorption spectroscopy.

The table shows the data used to determine the K_B values for GFT-M2@HSA.

GFT-M2@HSA (A0 = 0.13477)

1/[HSA]	A (340 nm)	Amax-A0/Ax-A0
166666.66667	0.15361	1.58526
125000	0.15539	1.44878
100000	0.15673	1.36
66489.3617	0.16023	1.17339
50000	0.16306	1.05577
39936.10224	0.16464	1

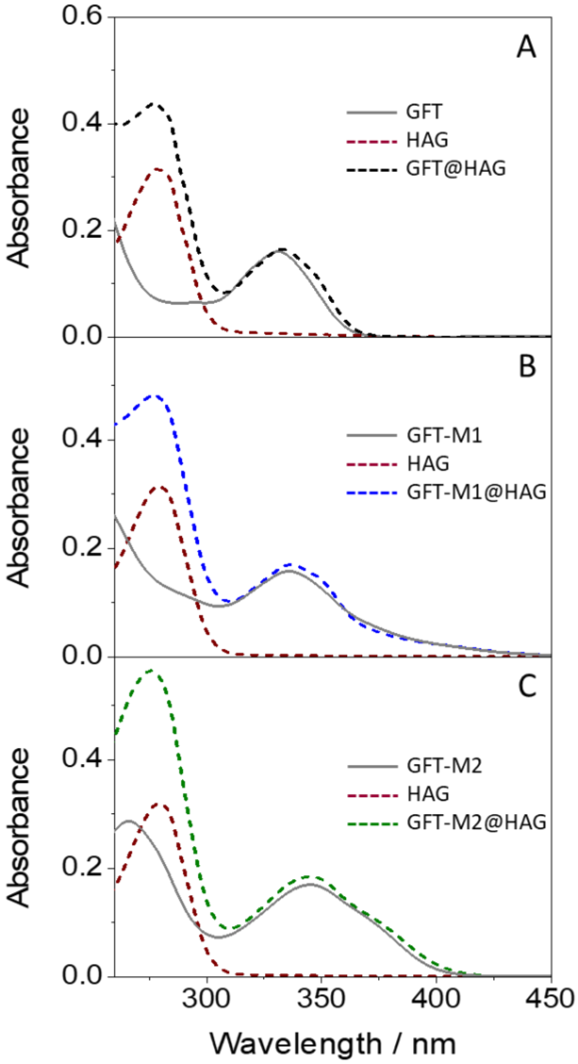

FIGURE S9. UV absorption spectra for (A) HAG (dashed dark red), GFT (gray) and GFT@HAG (dashed black), (B) HAG (dashed dark red), GFT-M1 (gray) and GFT-M1@HAG (dashed blue), and (C) HAG (dashed dark red), GFT-M2 (gray) and GFT-M2@HAG (dashed green). All measurements were performed at 10 μM in PBS. For ligand@HAG complexes, solutions were at 1:1 molar ratio.

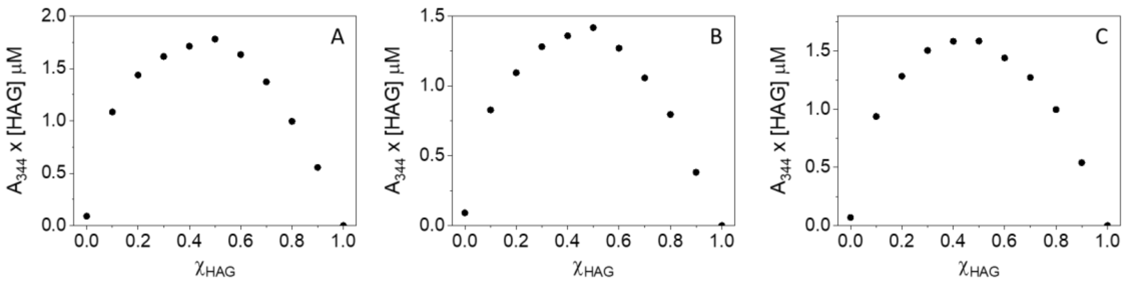

FIGURE S10. UV absorption Job's plot for (A) GFT@HAG, (B) GFT-M1@HAG, and (C) GFT-M2@HAG at a total concentration of 10 µM in PBS.

The tables show the values used for the Job's plot analysis.

GFT@HAG

χ_{HAG}	[HAG] (µM)	A332	[HAG]*A332
**0**	20	0.00447	0.08932
**0.1**	18	0.06019	1.08346
**0.2**	16	0.08977	1.43636
**0.3**	14	0.11526	1.61364
**0.4**	12	0.13851	1.66208
**0.5**	10	0.17802	1.7802
**0.6**	8	0.20397	1.63179
**0.7**	6	0.22862	1.37174
**0.8**	4	0.24873	0.99492
**0.9**	2	0.2775	0.55499
**1**	0	0.2976	0

GFT-M1@HAG

χ_{HAG}	[HAG] (µM)	A337	[HAG]*A337
**0**	20	0.00451	0.09023
**0.1**	18	0.0571	1.02784
**0.2**	16	0.08337	1.33388
**0.3**	14	0.10739	1.50349
**0.4**	12	0.11322	1.35869
**0.5**	10	0.14177	1.41766
**0.6**	8	0.15878	1.27026
**0.7**	6	0.1761	1.05662
**0.8**	4	0.19892	0.79568
**0.9**	2	0.1907	0.38139
**1**	0	0.19294	0

GFT-M2@HAG

χ_{HAG}	[HAG] (µM)	A344	[HAG]*A344
**0**	20	0.00344	0.06881
**0.1**	18	0.05206	0.93711
**0.2**	16	0.08022	1.28354
**0.3**	14	0.10746	1.50448
**0.4**	12	0.13191	1.58287
**0.5**	10	0.15858	1.58579
**0.6**	8	0.18004	1.44034
**0.7**	6	0.21204	1.27223
**0.8**	4	0.24903	0.99614
**0.9**	2	0.27005	0.54009
**1**	0	0.2916	0

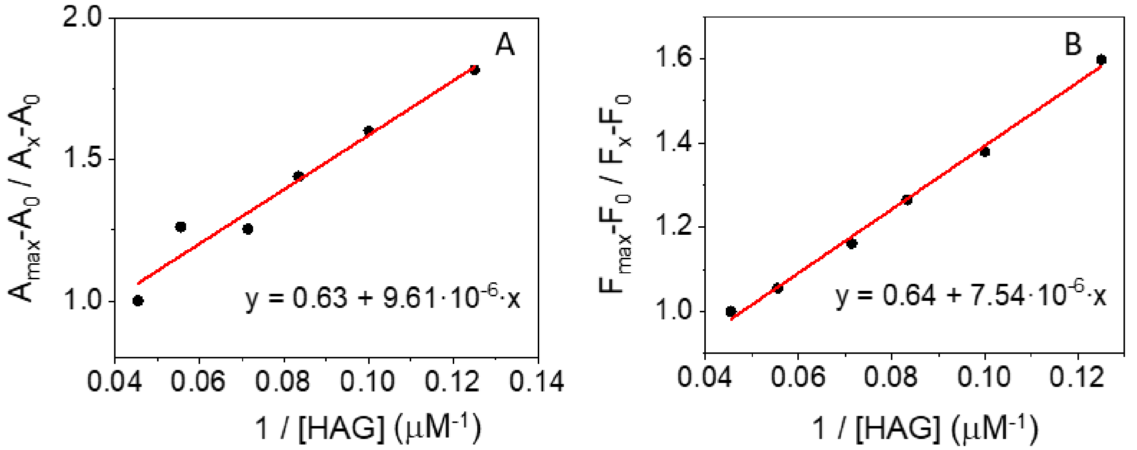

FIGURE S11. Linear fit of the modified Scatchard plot analysis to determine the K_B values for GFT@HAG by means of UV absorption (A) or fluorescence (B) spectroscopies.

The tables show the data used to determine the K_B values for GFT@HAG.

GFT@HAG (A0 = 0.08768)

1/[HAG]	A (340 nm)	Amax-A0/Ax-A0
125000	0.10101	1.81583
100000	0.1028	1.60042
83333.33333	0.10449	1.43963
71428.57143	0.10697	1.25443
55555.55556	0.10687	1.26123
45454.54545	0.11188	1

GFT@HAG (IF0 = 4935.33984)

1/[HAG]	IF (385 nm)	IFmax-IF0/IFx-IF0
125000	223126.156	1.59718
100000	257585.984	1.37933
83333.33333	280346.875	1.26534
71428.57143	305017.25	1.16132
55555.55556	335076.438	1.05558
45454.54545	353425.031	1

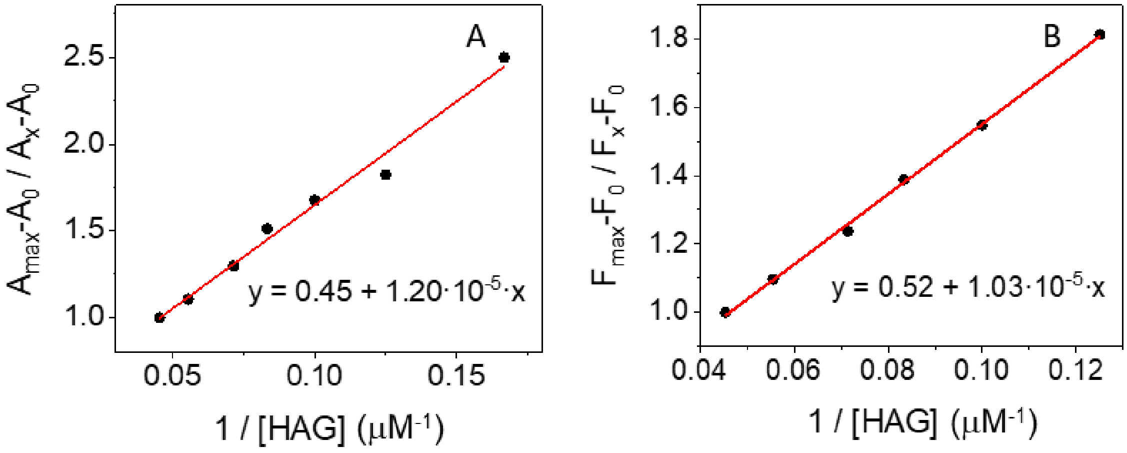

FIGURE S12. Linear fit of the modified Scatchard plot analysis to determine the K_B values for GFT-M1@HAG by means of UV absorption (A) or fluorescence (B) spectroscopies.

The tables show the data used to determine the K_B values for GFT-M1@HAG.

GFT-M1@HAG (A0 = 0.081294)

1/[HAG]	A (336 nm)	Amax-A0/Ax-A0
166666.66667	0.08848	2.49891
125000	0.09114	1.82277
100000	0.092	1.67632
83333.33333	0.09316	1.51223
71428.57143	0.09513	1.29708
55555.55556	0.09752	1.10579
45454.54545	0.09924	1

GFT-M1@HAG (IF0 = 7774.26172)

1/[HAG]	IF (445 nm)	IFmax-IF0/IFx-IF0
125000	157038.141	1.81276
100000	182656.672	1.54721
83333.33333	202690.016	1.38818
71428.57143	226529.422	1.2369
55555.55556	254654	1.096
45454.54545	278353.313	1

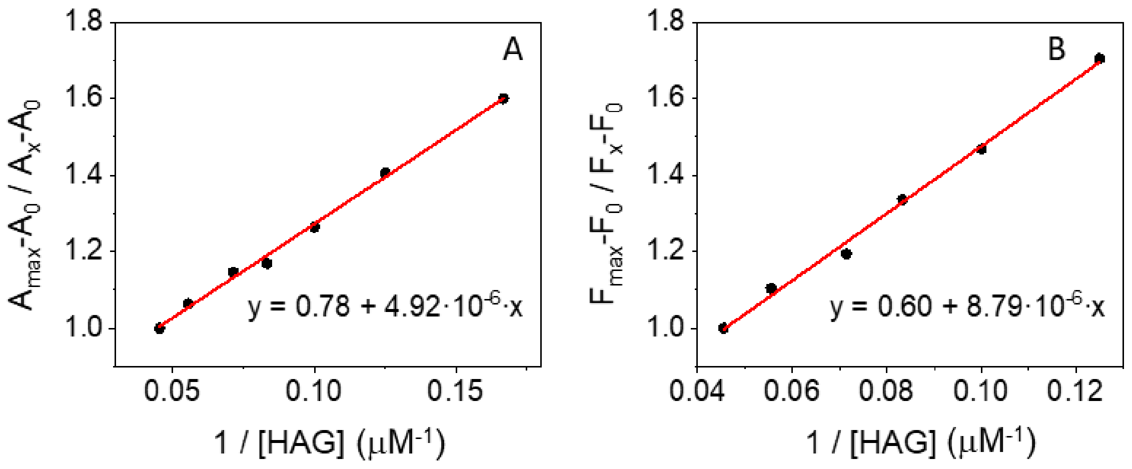

FIGURE S13. Linear fit of the modified Scatchard plot analysis to determine the K_B values for GFT-M2@HAG by means of UV absorption (A) or fluorescence (B) spectroscopies.

The tables show the data used to determine the K_B values for GFT-M2@HAG.

GFT-M2@HAG (A0 = 0.117185)

1/[HAG]	A (346 nm)	Amax-A0/Ax-A0
166666.66667	0.10689	1.60029
125000	0.10574	1.4056
100000	0.10481	1.26431
83333.33333	0.10456	1.16861
71428.57143	0.10359	1.14601
55555.55556	0.10272	1.06415
45454.54545	0.87654	0.99979

GFT-M2@HAG (IF0 = 8897.3418)

1/[HAG]	IF (450 nm)	IFmax-IF0/IFx-IF0
125000	158831.344	1.70497
100000	182984.063	1.46842
83333.33333	200232.578	1.33604
71428.57143	222972.141	1.19413
55555.55556	240499.406	1.10376
45454.54545	264529.625	1

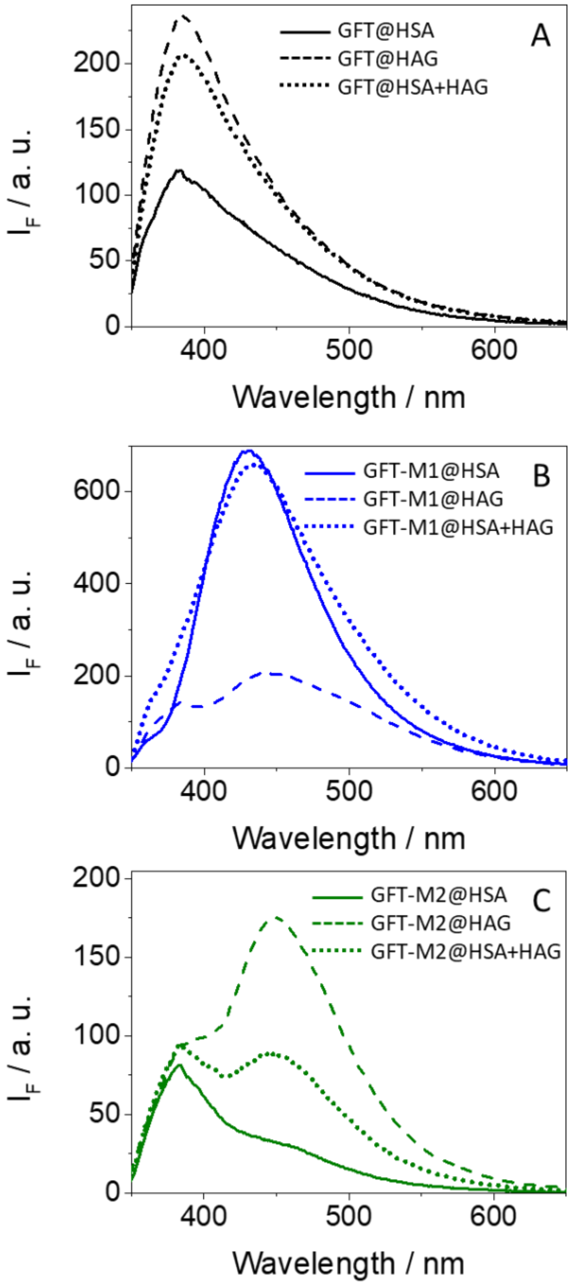

FIGURE S14. Fluorescence spectra at λ_{exc} = 340 nm for (A) GFT@HSA (solid black), GFT@HAG (dashed black) and GFT@HSA+HAG (dotted black), (B) GFT-M1@HSA (solid blue), GFT-M1@HAG (dashed blue) and GFT-M1@HSA+HAG (dotted blue), and (C) GFT-M2@HSA (solid green), GFT-M2@HAG (dashed green) and GFT-M2@HSA+HAG (dotted green). All mixtures were at 1:1 molar ratio (10 µM) in PBS. For drug (or metabolite)/HSA+HAG, equimolar 1:1:1 mixtures were used.

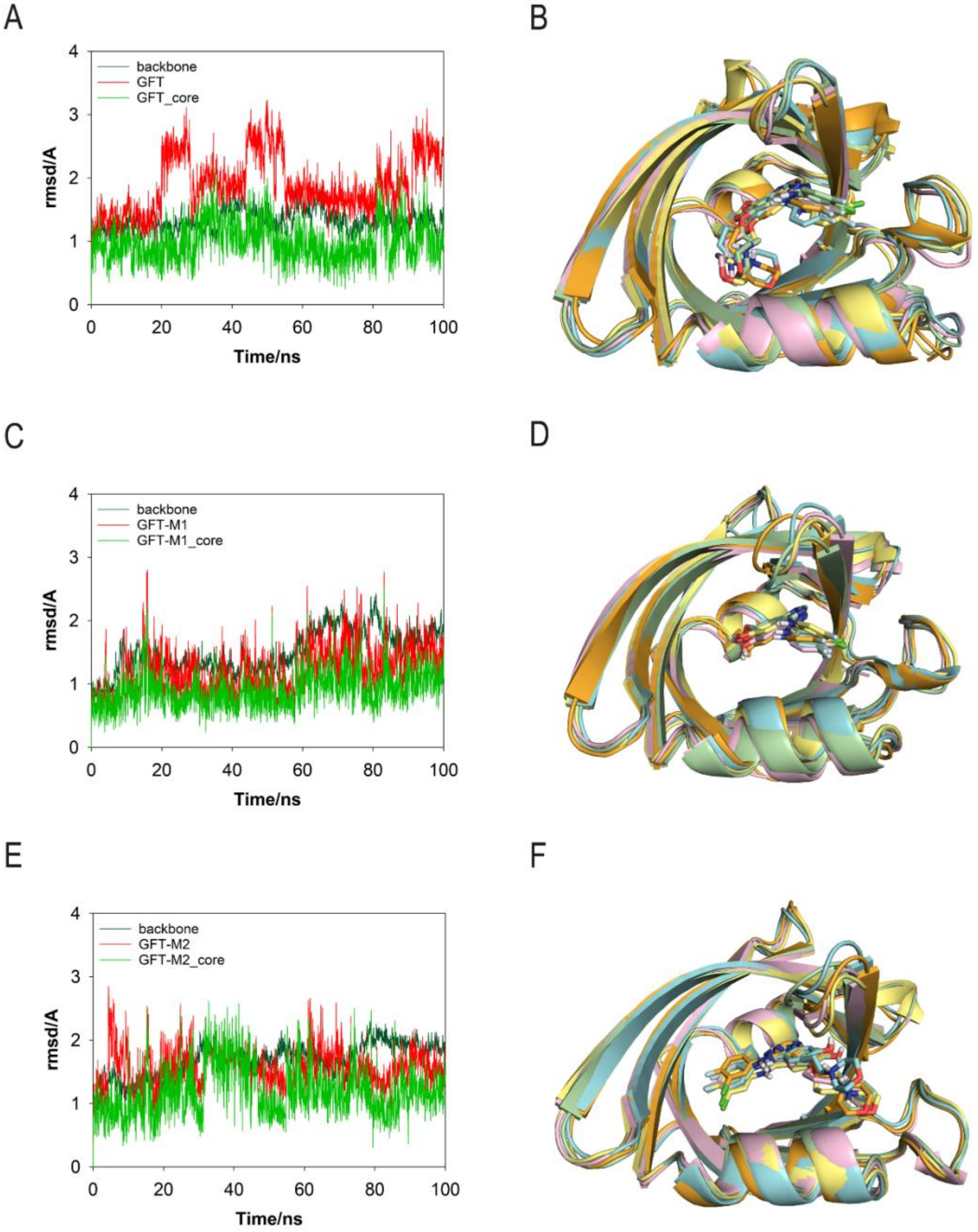

FIGURE S15. (A,C,E) RMSD plots for the protein backbone (Cα, C, O and N atoms), the ligand (all non-hydrogen atoms) and ligand core (only C and N atoms of the quinazoline moiety) calculated from the MD simulations of the complexes GFT@HAG (A), GFT-M1@HAG (B) and GFT-M2@HAG (C). (B,D,F) Comparison of several snapshots of the complexes GFT@HAG (B), GFT-M1@HAG (D) and GFT-M2@HAG (E) during 100 ns of MD simulation: at 20 ns (cyan), 40 ns (pale green), 60 ns (pale yellow), 80 ns (light pink) and 100 ns (orange). Ligands are depicted in sticks.

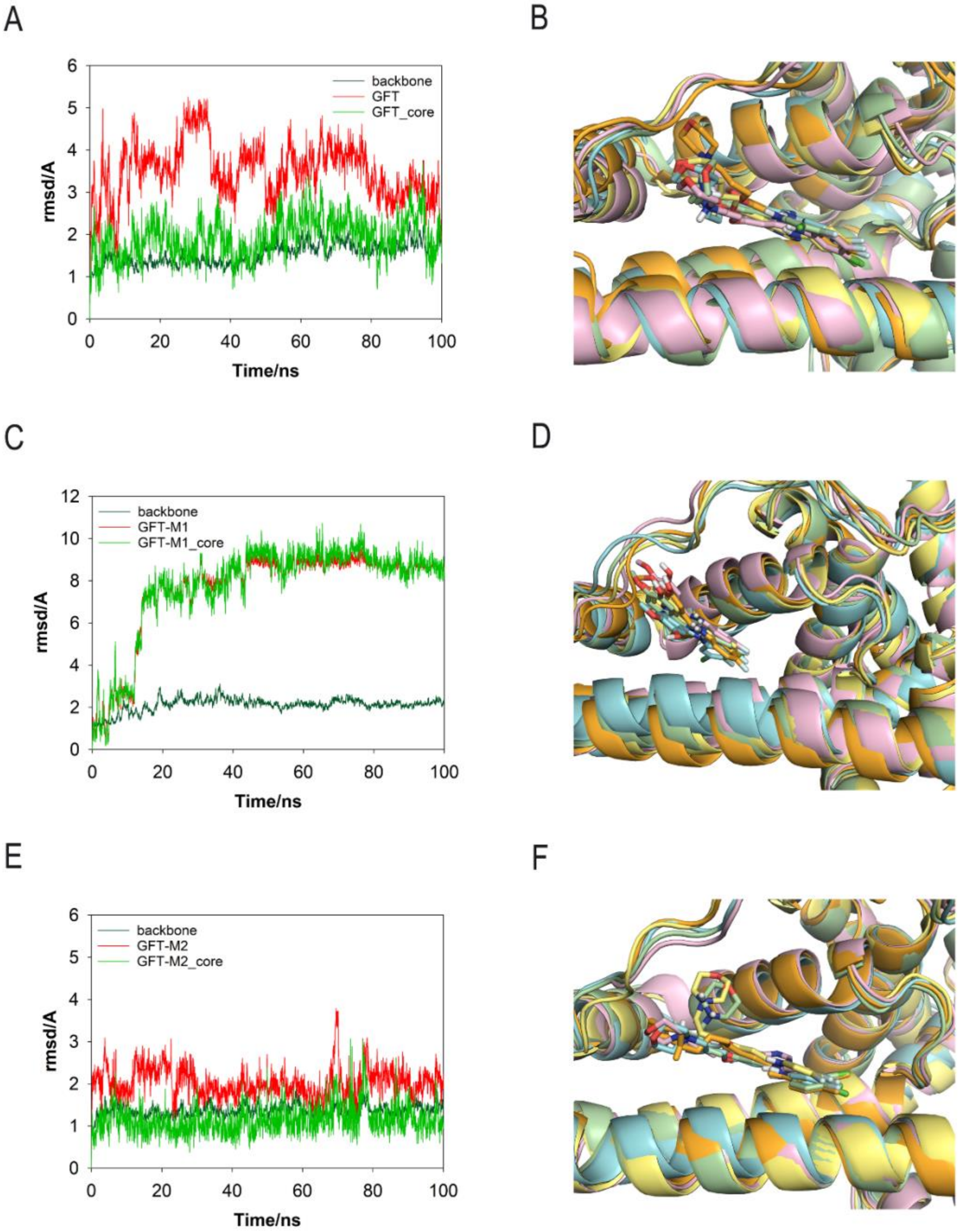

FIGURE S16. (A,C,E) RMSD plots for the protein backbone (Cα, C, O and N atoms), the ligand (all non-hydrogen atoms) and ligand core (only C and N atoms of the quinazoline moiety) calculated from the MD simulations of the complexes GFT@HSA (A), GFT-M1@HSA (B) and GFT-M2@HSA (C). (B,D,F) Comparison of several snapshots of the complexes GFT@HSA (B), GFT-M1@HSA (D) and GFT-M2@HSA (E) during 100 ns of MD simulation: at 20 ns (cyan), 40 ns (pale green), 60 ns (pale yellow), 80 ns (light pink) and 100 ns (orange). Ligands are depicted in sticks.
